# Robot-Based Procedure for 3D Reconstruction of Abdominal Organs Using the Iterative Closest Point and Pose Graph Algorithms

**DOI:** 10.3390/jimaging11020044

**Published:** 2025-02-05

**Authors:** Birthe Göbel, Jonas Huurdeman, Alexander Reiterer, Knut Möller

**Affiliations:** 1 Department of Sustainable Systems Engineering, NATECH, University of Freiburg, Emmy-Noether-Straße 2, 79110 Freiburg, Germany; alexander.reiterer@mail.inatech.uni-freiburg.de; 2 KARL STORZ SE & Co. KG, Dr.-Karl-Storz-Str. 34, 78532 Tuttlingen, Germany; jonas.huurdeman@karlstorz.com; 3 Institute for Bioinformatics and Medical Informatics (IBMI), University of Tübingen, Geschwister-Scholl-Platz, 72074 Tübingen, Germany; 4 Fraunhofer Institute for Physical Measurement Techniques IPM, Georges-Köhler-Allee 301, 79110 Freiburg im Breisgau, Germany; 5 Institute of Technical Medicine, ITeM, Furtwangen University (HFU), Jakob-Kienzle-Straße 17, 78054 Villingen-Schwenningen, Germany; knut.moeller@hs-furtwangen.de

**Keywords:** Image-based 3D reconstruction, laparoscopy, multi-view reconstruction, robot-assisted intervention, stereo vision

## Abstract

Image-based 3D reconstruction enables robot-assisted interventions and image-guided navigation, which are emerging technologies in laparoscopy. When a robotic arm guides a laparoscope for image acquisition, hand–eye calibration is required to know the transformation between the camera and the robot flange. The calibration procedure is complex and must be conducted after each intervention (when the laparoscope is dismounted for cleaning). In the field, the surgeons and their assistants cannot be expected to do so. Thus, our approach is a procedure for a robot-based multi-view 3D reconstruction without hand–eye calibration, but with pose optimization algorithms instead. In this work, a robotic arm and a stereo laparoscope build the experimental setup. The procedure includes the stereo matching algorithm Semi Global Matching from OpenCV for depth measurement and the multiscale color iterative closest point algorithm from Open3D (v0.19), along with the multiway registration algorithm using a pose graph from Open3D (v0.19) for pose optimization. The procedure is evaluated quantitatively and qualitatively on ex vivo organs. The results are a low root mean squared error (1.1–3.37 mm) and dense point clouds. The proposed procedure leads to a plausible 3D model, and there is no need for complex hand–eye calibration, as this step can be compensated for by pose optimization algorithms.

## 1. Introduction

Multi-view image-based 3D reconstruction describes the stitching of multiple depth maps of an object into a single 3D model. In laparoscopy, this could include the stitching of abdominal organs such as the liver or gallbladder. The advantage of multi-view reconstruction is that multiple perspectives of an organ can be observed, leading to a complete 3D model (rather than only parts of an organ), which is necessary for applications such as image-guided navigation and robot-assisted interventions. It requires two separate tasks: depth estimation and camera pose estimation. This article describes a procedure to achieve a complete 3D model of abdominal organs, with a focus on the second task, pose estimation. A robot delivers an initial guess for the camera pose, which is then optimized by two pose optimization algorithms. For the experimental setup, a stereo laparoscope and a robotic arm for camera guidance are utilized.

### 1.1. Related Work

In the literature, there are a few articles about multi-view reconstruction based on stereo laparoscopes. Reference [1] used stereo frames for depth estimation and combined optical tracking and an iterative closest point (ICP) algorithm for camera pose estimation. The accuracy was not measured. Similar to this, Reference [2] took calibrated stereo images and compared three different pose estimation algorithms, including simultaneous localization and mapping (SLAM), visual odometry (VO), and structure from motion (SfM), to reconstruct a phantom surgical cavity. Their findings indicated that SLAM is the most promising algorithm due to its fastest computation time and high accuracy and precision (a little less than SfM). For accuracy measurement, the root mean squared error (RMSE) was applied and resulted in 13.97 mm.

In contrast to these two studies, Reference [3] designed a stereo endoscope with two different states of stereo bases, which can be applied by pushing a button. By using a feature algorithm, they calculated a homography matrix to stitch together two monocular images taken from a bigger stereo base. Here, the accuracy was not measured. Reference [4] applied SLAM for camera pose estimation and a stereo matching algorithm for 3D reconstruction. In the end, they wanted to overlay augmented information during minimally invasive surgery (MIS). The measured RMSE for a simulated abdominal scene amounted to 2.37 mm.

Reference [5] proposed a method for 3D reconstruction based on a SLAM algorithm, in which feature patch extraction replaced feature point extraction, making the algorithm more robust. The method was validated on the laparoscopic in vivo Hamlyn dataset, and the RMSE varied between 5.2 mm, 2.3 mm, and 1.3 mm depending on the image resolution—the lower, the worse the RMSE. Reference [6] described the development of an autonomous scanning and mosaicking system with the help of the da Vinci ® surgical robot (Intuitive Surgical, Sunnyvale, CA, USA), an endomicroscope, and a stereo laparoscope. The camera motion estimation was performed by a tracking marker on the endomicroscope and the acquired images. There was no information offered about the 3D reconstruction accuracy.

Reference [7] used stereoscopy for 3D reconstruction and a 2D feature tracking algorithm for motion estimation to register the reconstructed points to a preoperative volumetric scan, reporting a registration error for intraoperative data of 1.6493 mm (error between points on the registered stereo reconstruction and the corresponding points on the preoperative volume). Reference [8] took stereo laparoscopic images and a SLAM algorithm called EMDQ-SLAM for 3D reconstruction, as well as camera motion and tissue deformation estimation. The accuracy was not computed.

### 1.2. Camera Pose Estimation by Uncalibrated Robot Position Combined with Pose Optimization Algorithms

Most of the aforementioned papers only focus on handheld laparoscopes, which is fine, as this is the state-of-the-art for most surgeons. However, the amount of robot-assisted surgery (RAS) increases yearly [9,10,11,12]. Research even gets a step further in the direction of autonomous robotic surgery as in [13,14,15]. In Reference [14], 3D reconstruction of the surgical scene is the basis for an autonomous suturing experiment. Thus, 3D reconstruction is an enabling technology for autonomy in RAS. Moreover, a robot-guided camera brings advantages to our 3D reconstruction approach, such as the precise execution of movements, the standstill during image acquisition, and the known robot position. Accordingly, this work presents a procedure to create a 3D model by utilizing the stereo laparoscope TipCam Rubina 1S 30° (KARL STORZ SE & Co. KG, Tuttlingen, Germany) guided by the UR5 CB3 robotic arm (Universal Robots A/S, Odense, Denmark).

When a camera is mounted on a robot’s flange, hand–eye calibration is usually executed to know the exact transformation between the camera and the robot frame [16]. There are different approaches to achieving this, and often such a process requires targets such as chessboard patterns or QR code-like patterns, making it complicated and error-prone [17]. Especially, surgeons and their assistant staff cannot be expected to execute it. Ref. [18] developed a hand–eye calibration routine without such printed targets but instead used the instruments during the intervention as targets. It resulted in a 3 mm accuracy of the 3D instrument positioning error, which might not be enough for a submillimeter-accurate 3D reconstruction. As discussed in [19], small measurement errors in the robot flange position lead to amplified errors at the laparoscope tip. Both the complicated hand–eye calibration process and the risk of errors motivate us to examine if hand–eye calibration can be left out and be substituted by plain image-processing or point cloud-processing software. Thus, the camera pose estimation does not rely solely on the robot but on the robot position combined with an ICP and a pose graph optimization algorithm.

## 2. Methods

The challenge of multi-view compared to single-shot 3D reconstruction is that not only the depth is estimated, but also these separate depth maps must be stitched together. Thus, two tasks can be separated:

1. Depth estimation for point cloud generation;
2. Camera pose estimation and optimization for point cloud stitching (the focus of this work).

**Figure 1:**
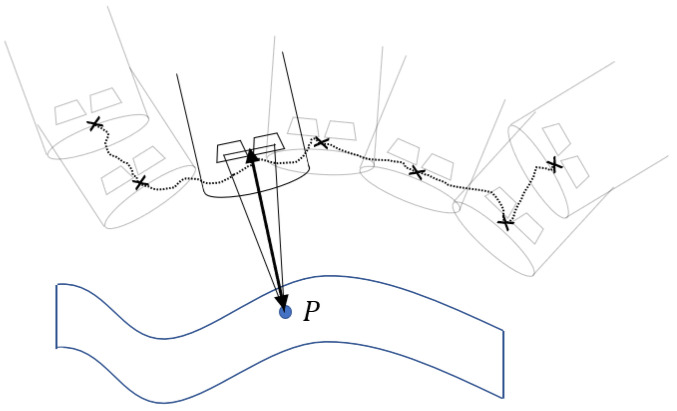
Schematic overview of the 3D reconstruction method stereoscopy. It shows the surface to be reconstructed, with point P in blue, the laparoscope tip with two image sensors generating the estimated depth (black arrow), and the estimated camera positions (x marks and dotted line).

There are different image-based methods to realize the first task, e.g., Structure-from-Motion (SfM), Shape-from-Shading (SfS), Time-of-Flight (ToF) cameras, and stereoscopy, as presented in [20]. In this work, stereoscopy is chosen for depth estimation (see Figure 1, showing the laparoscope tip with two image sensors). The reason is that compared to mono camera systems, the second camera delivers additional information, which enables 3D vision and accurate depth estimation as shown in [21]. Moreover, stereo camera systems are state-of-the-art systems for laparoscopic interventions [20].

The second task is relevant for aligning each single point cloud with its neighboring point cloud, leading to a complete merged point cloud in the end. This requires knowledge about all the passed camera positions (see Figure 1, black-dotted line). Ideally, the camera pose is known by a sensor, e.g. inertial measurement unit (imu), but standard laparoscopes do not include such and an imu suffers from drift, which leads to inaccurate poses. The alternative is a pose estimation algorithm based on image/point cloud processing or robotic guidance (where the camera position is known by the robot).

In this work, the laparoscope is guided by a robotic arm, which brings the advantage of knowing the robot flange position, and based on that, the camera position is calculated by applying a transformation matrix. For submillimeter accuracy, hand–eye calibration of the exact camera position relative to the robot flange would be necessary. As stated in the introduction, an alternative procedure is examined to avoid hand–eye calibration by instead utilizing two image-/point cloud-based algorithms for camera pose optimization, namely an ICP and a pose graph optimization algorithm. To show the effectiveness of this approach, three different stitching approaches based on the same stereo frames and the same robot positions are created and compared. The difference between each stitching is the method of pose estimation:

1. Rob only (only the uncalibrated robot positions are considered for stitching);
2. Rob + ICP (in addition to the robot position, the ICP algorithm is also applied for pose optimization);
3. Rob + ICP + pose graph (our final approach, which utilizes the uncalibrated robot position as an initial position guess alongside the ICP and pose graph algorithm for pose optimization).

The following chapter presents the experimental setup, the software architecture (including a detailed description of the ICP and pose graph optimization algorithm), and the evaluation of the results.

### 2.1. Experimental Setup

**Figure 2:**
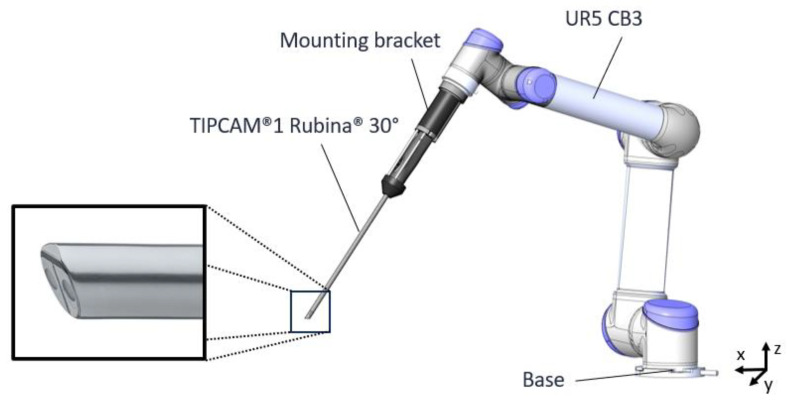
Schematic overview of the experimental setup. The TipCam Rubina 1S 30° is held by the UR5 CB3 robotic arm (Universal Robots A/S, Odense, Denmark). The video laparoscope is equipped with a stereo camera system with chip-on-the-tip technology, which is angled at 30° and has an 80° FOV.

The experimental setup includes the robotic arm UR5 CB3 (Universal Robots A/S, Odense, Denmark) for camera guidance, the stereo laparoscope TipCam Rubina 1S 30° (KARL STORZ SE & Co. KG, Tuttlingen, Germany) (see Figure 2) for image acquisition, and the EinScan H2 laser scanner (Shining 3D Tech Co., Ltd., Hangzhou, China) for ground truth acquisition. The stereo camera is angled at 30° and has an 80° FOV field-of-view (FOV). Each left and right image frame offers a Full HD resolution of 1920 × 1080 pixels, and image processing is partly run on an RTX A4000 graphics card (NVIDIA Corporation, Santa Clara, CA, USA). Ex vivo pig organs were used for the qualitative and quantitative evaluation. In this work, the fulcrum point—also known as the trocar point, which is the entry point of the laparoscope through the abdominal wall—is not considered. This constraint complicates the robotic movement for image acquisition because it reduces the six degrees of freedom (DOF) to four DOF. Thus, the robot is positioned so that the camera viewing direction is perpendicular to the object surface, and images are taken in a grid-shaped movement pattern. In total, 63 stereo frames are taken with a 70 mm distance between the laparoscope tip and the object and around 10 mm distance between neighboring image frames.

### 2.2. Architecture of the Robot-Based 3D Reconstruction Procedure

**Figure 3:**
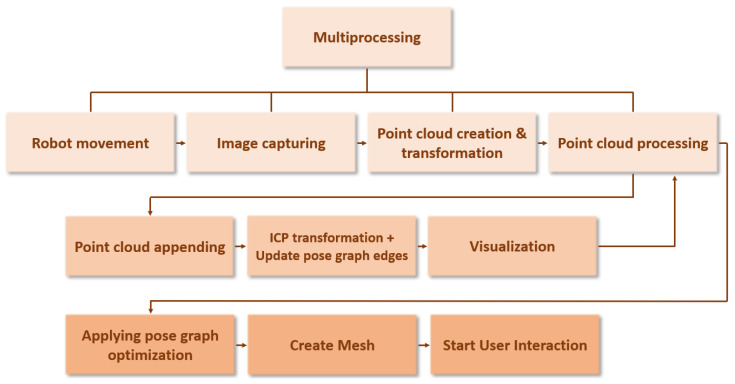
Visualization of the architecture of the robot-based 3D reconstruction procedure.

The procedure includes four parallel running processes: Robot movement, image capturing, point cloud creation and transformation (from camera coordinate system to world coordinate system), and point cloud processing (see Figure 3). The latter process includes the sub-processes point cloud appending, ICP computing and pose graph updating, and visualization. After point cloud processing, two postprocessing steps—pose graph optimization and mesh creation—are applied before user interaction begins. As soon as the robot reaches the first position, the first stereo frame is taken to be processed and visualized. As the robot moves on, more frames are taken, processed, and visualized. After the last frame is acquired and processed, postprocessing begins. This multiprocessing approach offers the advantage that the growing 3D model is already visualized during the robotic movement.

#### 2.2.1. Robot Movement

The robotic arm has a repeatability of ±0.1 mm and is controlled by Python via the real-time data exchange (RTDE) interface provided by Universal Robots A/S (Universal Robots A/S, Odense, Denmark). The command used for robotic movement is called movel (end_pose, a = 0.1, v = 0.05), which requires the target position, acceleration, and velocity as input parameters. The target position is a 6 DOF vector containing x-, y-, and z-coordinates and rx, ry, and rz as parts of the rotation vector. By default, this command works with respect to the base coordinate system. The movement in this experiment follows a grid-shaped pattern, which only changes in the x- and y-direction and is fixed in the z-direction (compare with the world coordinate system in Figure 2).

#### 2.2.2. Image Capturing

The images are captured as soon as the robot has reached its first resp. next target position and has come to a stop. Images are only taken if the robot stands still. For this experiment, the frame grabber USB Capture HDMI 4K Plus (Nanjing Magewell Electronics Co., Ltd., Nanjing, China) is used. The stereo camera has been calibrated to undistort each channel and align left to right.

#### 2.2.3. Point Cloud Creation and Transformation

Based on the calibrated stereo images, disparity maps are generated by the Semi Global Matching (SGM) algorithm with the command cv2.cuda.createStereoSGM() by OpenCV version 4.8.0, which can then be further converted into point clouds by the OpenCV command cv2.reprojectImageTo3D().

Depending on the robot trajectory, the object is seen from different perspectives, and for each perspective, a point cloud is generated. With the known robot position, these point clouds are stitched together, leading to a complete point cloud of the scanned object. The transformation from the robot flange to the camera is essential for stitching because, based on this information, each perspective’s camera position is estimated. The transformation from the camera tip position to the robot flange with respect to the robot base coordinate system involves a 180° turn around the y-axis (ϕ=180°), a −30° turn around the x-axis (θ=−30°), and a + 142° turn around the z-axis (ψ=142°), which creates the rotation matrix as in (1) and (2) and a translation in the z-direction of z=580 mm as in (3). These values arise from the CAD model (see Figure 2) and the OpenCV image frame convention, which defines the coordinate origin to lie in the upper left corner of the image, the x-axis pointing to the right and the y-axis pointing to the bottom. Due to the omitted hand–eye calibration, the transformation from camera to robot flange is only based on the known geometry of the mounting. This leads to uncertainties in the robot-based camera position estimation and errors in the stitched point cloud.

(1) $$R=R_{y,\phi }\times R_{z,\theta }\times R_{x,\psi }$$

(2) $$R=\left(\begin{array}{ccc} \cos\phi \cos\theta & -\cos\phi \sin\theta \cos\psi +\sin\phi \sin\psi & \cos\phi \sin\theta \sin\psi +\sin\phi \cos\psi \\ \sin\theta & \cos\theta \cos\psi & -\cos\theta \sin\psi \\ -\sin\phi \cos\theta & \sin\phi \sin\theta \cos\psi +\cos\phi \sin\psi & -\sin\phi \sin\theta \sin\psi +\cos\phi \cos\psi \end{array}\right)$$

(3) $$t=\left(\begin{array}{c} 0\\ 0\\ z \end{array}\right)$$

#### 2.2.4. Point Cloud Processing

As said before, the missing hand–eye calibration causes positional errors in the 3D model. Consequently, point cloud processing includes an ICP algorithm with the purpose of an optimized surface alignment between neighboring point clouds. The algorithm used in this work is the multiscale colored ICP algorithm from Open3D (v0.19) (cuda compatible) and is based on the colored ICP registration algorithm from Reference [22], including both geometry and color information by computing a joint photometric and geometric optimization objective (o3d.t.pipelines.registration.multi_scale_icp()). The goal is to find a transformation T that aligns two colored point clouds by taking the colors (photometric term $E_{C}$) and the geometry (geometric term $E_{G}$) as well as a weight $\sigma \;\in \left[0,\;1\right]$ into account, as in (4) [22].

(4) $$E\left(T\right)=\left(1-\sigma \right)E_{C}(T)+\sigma E_{G}(T)$$

A further pose optimization is performed by the multiway registration algorithm via pose graph by Open3D (v0.19) with the command o3d.pipelines.registration.PoseGraph(), which is based on References [23,24]. It is applied when multiple point clouds must be aligned in a global space, and it tackles the challenge of pruning incorrect transformations by modeling the validity of loop closure pieces with low overlap. The pose graph algorithm defines nodes and edges, from which in our case the nodes contain the position information coming from the robot, and the edges contain the transformation information computed by the ICP. The edges act as constraints between the robot poses (nodes). The goal of the algorithm is to find a configuration of the nodes that is consistent with the information in the edges. After each pairwise ICP transformation, the pose graph edges are updated with this information. The final step of the pose graph algorithm is executed in the postprocessing step because it is necessary that all positions of the robot trajectory have been reached to connect not only neighboring and overlapping point clouds but also nodes of non-overlapping areas. After ICP transformation and edge updating, the growing point cloud is visualized.

#### 2.2.5. Postprocessing

When all images are acquired, the visualized point cloud is closed, and postprocessing starts. At this stage, pose graph optimization is executed by the command o3d.pipelines.registration.global_optimization(), which requires the previously created map containing nodes and edges, as well as the optimization method based on the Levenberg–Marquardt algorithm, which is known for a faster convergence compared to the Gauss–Newton method. The global optimization is performed in two steps: the first one considers all edges, and the second step prunes uncertain edges. After this, the point cloud is finally transformed into a mesh by the Open3D (v0.19) command o3d.geometry.TriangleMesh.create_from_point_cloud_poisson() based on Reference [25], which generates a watertight closed surface. It includes the color information, which is stored in each point cloud and is transferred from there to every vertex (point building a triangle) in the mesh. The colors between vertices are interpolated, which leads to a smooth color distribution and reduces light reflections and non-uniform illuminations from the captured images. Finally, the mesh is displayed to the user and ready for use.

### 2.3. Qualitative and Quantitative Evaluation of 3D Reconstruction

The qualitative evaluation focuses on a dense point cloud with fewer holes, color fastness, and a plausible and steady topology. The quantitative evaluation focuses on the root mean squared error (RMSE) of the reconstruction error, which is the distance between 3D points on the reconstructed surface ${\hat{y}}_{i}\;$and the corresponding points on the real surface $y_{i}$ in mm, as in (5), with N representing the number of reconstructed points. The reconstruction error is measured by aligning the reconstructed surface to the ground truth surface, first manually and then by applying the ICP for fine-tuning.

(5) $$RMSE=\sqrt{\frac{1}{N}\sum_{i=1}^{N}{\left({\hat{y}}_{i}-y_{i}\right)}^{2}}$$

## 3. Results

**Figure 4:**
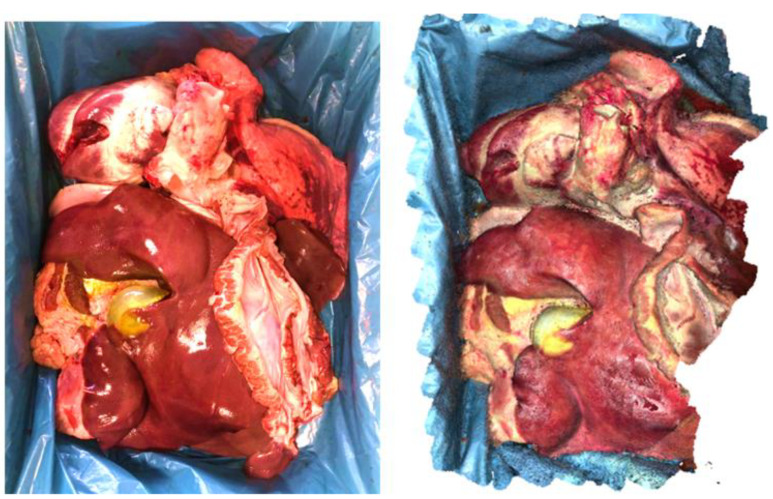
Photography of pig organs (**left**) and screenshot of the reconstructed point cloud created by our approach (**right**).

**Figure 5:**
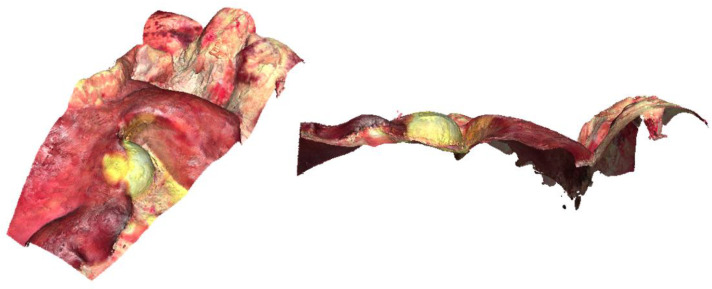
Screenshot of a section of the reconstructed point cloud in front view (**left**) and side view (**right**).

The final experimental results are visualized as point clouds and as mesh (closed surface) by the 3D mesh processing software MeshLab (Instituto di Scienza e Tecnologie dell’Informazione, San Giuliano Terme, Italy). The qualitative results show a dense point cloud (see Figure 4 (right) and Figure 5) with plausible topology and colors when compared with the photography (see Figure 4 (left)).

**Figure 6:**
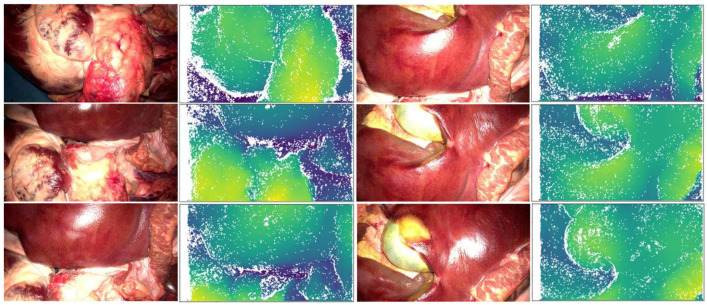
Six example images from the examined dataset (always left images) (**left**) and the corresponding depth maps with nan values in white, closer objects in more yellowish tones, and objects further away in more blueish colors (**right**).

The first task, “depth estimation for point cloud generation”, is performed by the stereo matching algorithm SGM from OpenCV, and a part of the results can be seen in Figure 6. There, six example images (always the left image) are presented showing excerpts of the heart, liver, and gallbladder, and for each image, the corresponding depth map is shown. The colors in the depth maps represent the depth with nan values in white, closer objects in more yellowish tones, and objects further away in more blueish colors. The depth maps are mostly dense except for areas that are hidden from one of the two cameras or areas with reflections.

**Figure 7:**
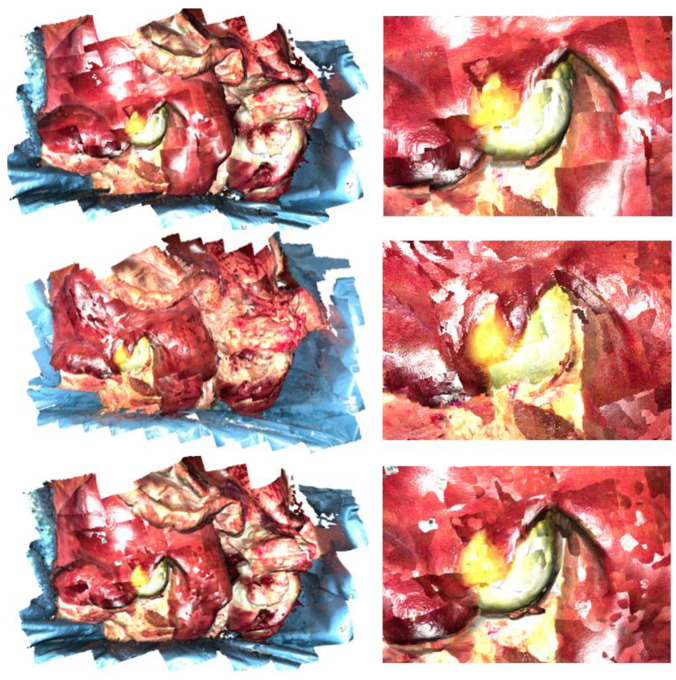
Screenshots of the pig organ point clouds (**left**) and an excerpt with a focus on the gallbladder (**right**) created by three different approaches: camera position estimation only by robot kinematics (Rob only) (**top**), by robot kinematics + ICP (Rob + ICP) (**middle**), by robot kinematics + ICP + pose graphs (Rob +ICP + pose graph) (**bottom**).

The second task, “camera pose estimation and optimization for point cloud stitching”, is performed by first transforming each point cloud based on the known robot position and second by applying the ICP and pose graph algorithm. To show their effectiveness, three different approaches are distinguished: Rob only, Rob + ICP, and Rob + ICP + pose graphs. The corresponding resulting point clouds are presented in Figure 7. The Rob only approach results in a stitched point cloud with edges between neighboring point clouds, which do not fit very well (see an excerpt of the gallbladder in Figure 7 (top right)). The additional use of the ICP improves the transitions between neighboring point clouds but lacks the consideration of an overall position optimization (see Figure 7 (middle)). The reconstructed gallbladder is not as round as it is. Thus, the pose graph algorithm ensures further position optimization, which leads to an improved stitched point cloud (see Figure 7 (bottom)). Here, the reconstructed gallbladder is round, the transitions between the neighboring point clouds are smooth, and no edges can be observed.

Compared to the point clouds seen in Figure 4 and Figure 5, the point clouds in Figure 7 miss any filtering regarding the reflections. Moreover, the brightness distribution within each single point cloud corresponds with the original colors of the image frames. There, the corners of the image are darker than its center. The reflections are reduced by filtering out those pixels with values over 0.97 (when the range for R, G, and B is from zero to one). As mentioned in Section 2, the mesh function of Open3D (v0.19) interpolates colors between vertices, leading to a smooth color distribution.

**Figure 8:**
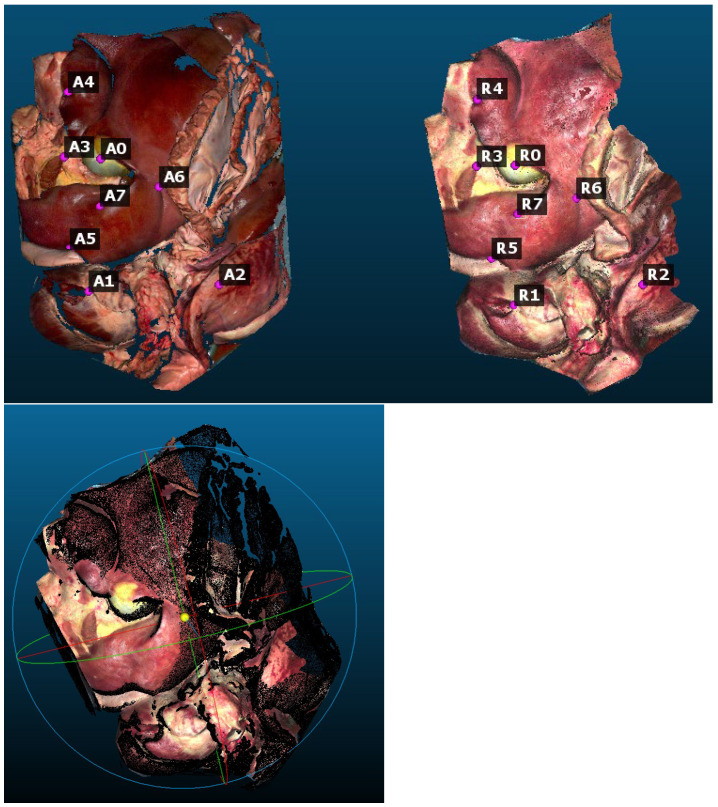
Screenshot of the ground truth point cloud (**left**) and the reconstructed point cloud by our approach (**middle**) with markers A0–A7 and R0–R7. The markers are used for point cloud alignment to compute the reconstruction error as RMSE. Screenshot of the overlaid ground truth in black and the reconstructed point cloud in colors to compute the reconstruction error (**right**).

The quantitative results are generated with the software CloudCompare 2.14.alpha (www.cloudcompare.org). The reconstruction error is calculated by overlaying the reconstructed point cloud onto the ground truth point cloud, on which the eight markers A0 to A7 on the ground truth and R0 to R7 on the reconstructed point cloud are selected for alignment (see Figure 8). Afterward, the ICP is applied for RMSE computation. The RMSE value is 3.37 mm for the whole point cloud seen in Figure 8 and Table 1 (Rob + ICP + pose graph), while the RMSE value is 1.1 mm for the excerpt seen in Figure 5. In comparison to that, the RMSE value for Rob only is 6 mm, and for Rob + ICP without pose graph, it is 4.37 mm, which shows the improvement caused by the pose graph algorithm.

## 4. Discussion

This work examined the impact of pose optimization algorithms on the appearance and accuracy of stitched point clouds. The main achievement and contribution of this work is that the pose estimation error, caused by an uncalibrated robot-guided camera system, can be corrected successfully by the Open3D (v0.19) pose optimization algorithm multiscale colored ICP and the multiway registration algorithm via pose graph. This can be observed in Section 3 (Figure 5). The results show that the combination of robotic camera guidance with ICP and pose graph algorithms (Rob + ICP + pose graph) leads to an accurate and realistic-looking 3D model of ex vivo organs. The appearance is similar to the ground truth point cloud and the photograph. The accuracy of 1.1–3.37 mm is within the range of other approaches dealing with ex vivo organs. Ref. [21] is a review focusing on the reconstruction error with the finding that the error for ex vivo organs lies between 1.1 and 10.8 mm (RMSE), where some references only focused on a single shot and some on multi-view reconstruction. Thus, our approach can compete with approaches with a similar setup.

In this work, only ex vivo organs are used for validation. Open-source in vivo data, including stereo images and robotic poses, do not exist, and an animal test is not yet possible, which leads us to validate our approaches on ex vivo organs. The difference between in vivo and ex vivo data is mainly the occurrence of instruments, blood, smoke, and movements (breathing and intestinal peristalsis) in vivo. Instruments, blood, and smoke will not be a problem in our case because the plan is to offer the 3D model as an assistive tool for the surgeon during the intervention or to use it for autonomous camera guidance, which means image acquisition must occur right before the surgeon starts the intervention. The two things that happen in vivo and affect our 3D reconstruction are movements emerging from breathing and intestinal peristalsis. Breathing takes place at a certain frequency, which must be detected to compensate for it. If detected, images could be taken directly after the exhalation cycle (empty lungs) or inhalation cycle (full lungs). That would lead to two 3D models—fully inhaled and fully exhaled. This must be examined in our next experiment and could be simulated with ex vivo organs by implementing a motor that generates a breathing-like movement. A potential solution to avoid breathing movements is to stop the breathing of the patient during image acquisition. If permitted by the medical staff, the time for the whole organ scan must be reduced significantly to around 10–20 s. The occurrence of intestinal peristalsis is not predictable and leads to errors in the stitching procedure (excessive movement and neighboring point clouds not matching). The scan must be repeated.

In summary, for a final validation of our approach, an animal lab is required for in vivo data. But for the current project state, the validation on ex vivo data is sufficient and more ethically defensible.

According to Ref. [21], a standard validation scenario should be set up to compare our approaches with those of other researchers. Because of this, the collected image data of the ex vivo organs including the corresponding robot positions and the ground truth point cloud of this experiment can be obtained from the authors. Ex vivo organs can already give an indication of whether an approach works successfully in a real interventional scenario, but in vivo data will additionally be required to critically validate an approach. Thus, our approach must be validated in an animal lab to address challenges such as movements (heartbeat, breathing, and intestinal peristalsis).

The fact that the fulcrum point is not considered in this work is a critical limitation. The fulcrum point complicates adopting the ideal perspectives (perpendicular) from the camera onto the tissue because it decreases the laparoscopic movement from six to only four DOF [26]. To achieve coverage of all areas of the tissue, the laparoscope’s motion must be a combination of rotation around its longitudinal axis and pivoting around the fulcrum point. In this case, the translation of the camera position between neighboring frames consists of rotation and translation related to various axes. In comparison to that, the omission of the fulcrum point allows a linear camera motion following only translational changes in the x- and y-direction.

Moreover, reflections disturb the quality of the resulting point cloud, and filtering is necessary. Also, the illuminance of the laparoscope’s light decreases with increasing distance (following the inverse square law for illuminance) [27], which leads to a difference in brightness within each frame. The result is darker colors in the image corners and brighter colors in the image center, which also degrades the quality of the resulting point cloud (mosaic-like appearance). However, not only the inverse square law for illuminance causes the brightness difference, but also the image acquisition from different perspectives and different working distances (distance between laparoscope tip and object). This leads to different color appearances of the objects. This must be fixed by filtering.

Currently, the whole procedure takes three minutes depending on the number of images. Most of the time is consumed by the initialization of the robot and the script (~30 s), the robot’s movement (~400 ms between frames), and the ICP and pose graph optimization (~510 ms per frame pair). The next improvement for latency reduction is the installation of a PCI express frame grabber by Magewell (Nanjing Magewell Electronics Co., Ltd., Nanjing, China), which should reduce the current time (~250 ms) to grab a stereo frame.

## 5. Conclusions

This work presents a procedure to achieve a complete 3D model of the abdomen and the internal organs. It requires a robotic arm and a stereo laparoscope for image acquisition and pose estimation. The depth estimation is based on a stereo matching algorithm and the camera pose estimation is a combination of the robot position and the optimization algorithms ICP and multiway registration via pose graph—both provided by Open3D (v0.19). For validation, ex vivo organs, including the heart, liver, gallbladder, and lung, were collected along with a laser scanner for ground truth acquisition. The qualitative results show dense point clouds, and the quantitative results show a high accuracy of 1.1–3.37 mm (RMSE of the reconstruction error) depending on the removal of outliers and the region of interest (the larger the reconstructed area, the higher the reconstruction error).

The next steps will be the automatic trajectory planning and execution based on a rough quick scan directly after laparoscope insertion (only a few millimeters inside the abdomen), the automatic detection of holes in the 3D model as well as a method to fill the holes, the improvement for real-time computation, and a proposal on how the surgeon can apply the 3D reconstruction as an interactive 3D model to automatically guide the laparoscope to a certain region of interest.

## Figures and Tables

**Table 1:** Overview of the quantitative evaluation by computing the RMSE of the reconstruction error depending on the pose estimation method: Rob only, Rob + ICP, and Rob + ICP + pose graph.

Pose Estimation Method	RMSE in mm
Rob only	6.0
Rob + ICP	4.37
Rob + ICP + pose graph	3.37

## Data Availability

The data that supports the findings of this work are available from the corresponding author.
